# CT perfusion lesions are present in most MRI confirmed lacunar strokes

**DOI:** 10.1016/j.nicl.2025.103903

**Published:** 2025-11-06

**Authors:** James O. Thomas, Milanka Visser, Carlos Garcia-Esperon, Neil J. Spratt, Dennis Cordato, Cecilia Cappelen-Smith, Longting Lin, Mark W. Parsons

**Affiliations:** a South Western Sydney Clinical School, University of New South Wales, Liverpool, NSW, Australia; b Department of Neurology, John Hunter Hospital, New Lambton Heights, NSW, Australia; c College of Health, Medicine and Wellbeing, University of Newcastle, Callaghan, NSW, Australia; d Hunter Medical Research Institute, New Lambton Heights, NSW, Australia; e Melbourne Brain Centre, University of Melbourne, Parkville, VIC, Australia; f Department of Neurophysiology, Liverpool Hospital, Liverpool, NSW, Australia

**Keywords:** Cerebral small vessel disease, Lacunar stroke, Perfusion imaging

## Abstract

Lacunar stroke remains diagnostically challenging in acute settings due to limited sensitivity of routine imaging. While CT perfusion (CTP) is widely used for evaluating stroke, its role in the identification of subcortical lacunar infarcts is less clear. Current core/penumbra algorithms used for CTP interpretation intentionally suppress small subcortical lesions rendering them unsuitable for lacunar stroke diagnosis. This retrospective, multicentre study evaluates the frequency of perfusion abnormalities in retrospectively confirmed lacunar stroke cases and characterizes clinical and radiological differences between cases with and without perfusion abnormalities.

We reviewed consecutive patients presenting to two comprehensive stroke centres between 2018 and 2022, who underwent acute multimodal CT and had a final diagnosis of acute lacunar stroke confirmed by diffusion-weighted MRI. Patients were included if no perfusion lesion was identified on algorithmic core/penumbra maps. CTP parameter maps were reviewed for the presence of focal perfusion abnormalities by stroke neurologists aware of the clinical scenario but blinded to MRI findings, followed by a second review with DWI co-registration to identify additional subtle perfusion abnormalities.

Of the 183 patients meeting inclusion criteria, 107 (58 %) demonstrated obvious perfusion abnormalities on blinded CTP review, an additional 36 (20 %) were found to have subtle lesions identified retrospectively with DWI guidance, resulting in an overall frequency of 78 %. Cases with no perfusion abnormality (22 %) had significantly smaller infarct volumes (median 0.7 ml vs 1.8 ml, p < 0.001), lower baseline NIHSS scores (median 2 vs 4, p = 0.005), and longer time from onset to imaging (median 8.4 h vs 4.5 h, p = 0.033).

Despite negative algorithmic CTP maps being negative, in DWI proven lacunar stroke, focal perfusion abnormalities are in fact present in most cases. CTP-negative cases appear to represent a distinct subgroup with smaller infarcts and milder deficits. These results highlight the influence of infarct volume and scan timing on lesion visibility and suggest that with advanced processing or machine learning assisted interpretation, CTP could play a greater role in the acute assessment of lacunar stroke.

## Highlights

• Focal perfusion abnormalities are present in 78% of all lacunar strokes.
• Low signal to noise ratio on subcortical CT perfusion limits interpretation.
• Brainstem perforator strokes are less likely to be seen on CT perfusion.
• Perfusion-negative lacunar strokes tend to be smaller and less severe.

## 1 Introduction

Ischemic stroke is the second leading cause of death, and third leading cause of disability worldwide (Feigin et al., 2021). Advances in acute stroke care, with the development of intravenous thrombolysis and endovascular thrombectomy, have significantly improved outcomes for patients with large or medium vessel occlusion (LVO or MeVO), where a vessel occlusion is readily identified on imaging.

In contrast, lacunar stroke, caused by occlusion of small perforating arterioles, remains diagnostically challenging. These vessels lie below the resolution of standard CT angiography, making them invisible on routine vascular imaging. While diffusion-weighted MRI (DWI) can reliably confirm acute lacunar infarcts, access is often limited in the hyperacute phase, particularly outside tertiary centres. As a result, diagnosis frequently relies on clinical assessment alone, and can be inaccurate (Gerraty et al., 2002). The natural imaging history of lacunar syndrome can range from no long-term MRI lesion (up to 24 %) (Doubal et al., 2011), development of subcortical white matter hyperintensities, or development of a classical lacune (Wardlaw et al., 2013). Similar variability is seen in the clinical outcomes, with a majority of patients remaining independent at 12 months, though a significant minority (34 %) suffer long term disability (Bamford et al., 1987).

Multimodal CT (mCT), combining non-contrast CT, CT angiography, and CT perfusion (CTP), is increasingly used in acute stroke management. Though there is considerable variability internationally, in Australia and New Zealand, there is reasonably high penetration of routine CTP, even in smaller district hospitals (Garcia-Esperon et al., 2023). CTP is used to quantify brain tissue at risk and irreversibly damaged tissue to improve access to reperfusion therapies. CTP also improves accuracy of acute stroke diagnosis by highlighting areas of subtly reduced tissue perfusion (Campbell et al., 2013, Arora et al., 2022). There is evidence to suggest that this may also be true in the case of perforating arteriole occlusion or lacunar stroke (Garcia-Esperon et al., 2021:). However, the diagnostic performance of CTP in lacunar stroke is less well established. Prior studies have identified focal perfusion abnormalities in lacunar infarcts, particularly on transit time maps such as Tmax or delay time, but reported sensitivity remains low (0–62.5 %), with significant methodological variation (Garcia-Esperon et al., 2021, Thierfelder et al., 2014, Chiu et al., 2015, Das et al., 2015, Rudilosso et al., 2015, Benson et al., 2016, Cao et al., 2016, Tan et al., 2016). With rapid advances in imaging technologies and automated imaging interpretation with artificial intelligence algorithms, highly technical imaging analysis such as this has the potential to not only become far more accessible but raises the potential of greater clinical insights to be derived from current imaging. The high resolution, 4-dimensional imaging of CT perfusion has the potential to provide far more clinical information than is currently provided by the perfusion maps.

There are two main hypotheses for the modest sensitivity of CTP in this context. First, technical limitations such as low spatial resolution and signal-to-noise ratio may hinder detection of small-volume lesions (González, 2012). Our experience is that this is heavily influenced by the experience of the reporting clinician. Secondly, the pathophysiology of lacunar stroke is heterogeneous; mechanisms such as lipohyalinosis, branch atheromatous disease, or embolism may yield perfusion lesions with varying size, shape and location (Yaghi et al., 2021). The deep white matter tracts are highly eloquent structures with sub-millimetric differences in lesion size or location potentially causing marked differences in clinical deficits. There is also variation in the natural history of lacunar stroke, with spontaneous reperfusion being well described in lacunar stroke, both histopathologically and radiographically, which would be expected to significantly alter perfusion characteristics (Fisher, 1979, Shahi et al., 2014).

We sought to describe the frequency of perfusion abnormalities on CTP in a cohort of DWI confirmed lacunar stroke, and to describe the clinical and radiological characteristics of cases with and without abnormal perfusion on initial CTP scans. We hypothesized that significantly more strokes would be identified on retrospective review of the imaging, and that the strokes that are truly undetectable on CTP will have smaller infarct volume and be less severe.

## 2 Materials and methods

### 2.1 Study design

Our study followed a retrospective observational cohort design to compare the clinical and radiological characteristics of lacunar strokes with and without perfusion abnormalities on initial CTP scan.

### 2.2 Study population

We retrospectively analysed consecutive acute stroke cases presenting to two comprehensive stroke centres located in New South Wales, Australia, between 2018 and 2022 undergoing acute mCT.

Adult patients presenting with a clinical syndrome compatible with acute stroke and investigated with acute CTP were screened for inclusion. Patients were included in the study if no vessel occlusion or software defined perfusion lesion (as defined by the automated core-penumbra maps) was identified on acute multimodal CT imaging. The current algorithms used in all recruiting sites are programmed to exclude all any lesions below a pre-specified volume, essentially preventing automated identification of lacunar stroke on core-penumbra maps. Cases were excluded if the final clinical diagnoses recorded in the medical record did not include ischemic stroke and/or no lesion was identified on follow-up DWI.

### 2.3 Ethics

Patients were recruited as part of the INternational Stroke Perfusion Imaging Registry (INSPIRE). Human ethics approval was granted by the Hunter New England Human Research Ethics committee and local hospital ethics committees in accordance with Australian National Health and Medical Research Council guidelines.

### 2.4 Data collection

Following case ascertainment, clinical data were collected through retrospective review of the medical record. CTP was captured using several different scanners (Toshiba Aquillion One, Siemens Somatom Force, Siemens Definition AS+, GE Revolution CT). However, all was processed using MIStar software (Apollo Medical Imaging Technology, Melbourne, Australia) using the smallest slice thickness data available with no downsampling. Cerebral blood flow (CBF), cerebral blood volume (CBV), mean transit time (MTT) and delay time (DT) maps were exported and anonymized. Clinical presentation, risk factor, treatment metrics, and outcome data were obtained from the medical record.

### 2.5 Lacunar lesion detection on CTP

The CTP maps were reviewed by stroke neurologists (JT, CGE, MP), with 5–20 years of experience with mCT, who were provided description of clinical symptoms of each patient. The lacunar lesion detection on CTP included the following two steps:

Step 1: The imaging readers were blinded to follow-up DWI. All CTP maps were reviewed concurrently and the presence and location of an imaging abnormality thought to represent an acute stroke was recorded for NCCT, CBF, CBV, MTT and DT maps individually. Disagreements were resolved by majority consensus of the three readers.

Step 2: Following blinded assessment, the imaging readers reviewed CTP maps alongside follow-up DWI. The DWI image was registered into the same orientation as the CTP images using an in-house algorithm. Each case was reviewed for the presence of a focal, asymmetric perfusion abnormality. Similarly to step one, any disagreement was resolved by majority consensus.

### 2.6 Lacunar lesion detection on DWI

After all CTP images were reviewed, all DWI scans were reviewed by a stroke neurologist (JT), to determine the infarct topography and presumed culprit vessel (single anterior lenticulostriate, multiple anterior lenticulostriate [striatocapsular], thalamoperforator, cortical perforator, basilar perforator, artery of Percheron, anterior circulation multi-embolic, posterior circulation multi-embolic). The culprit vessel was determined by reviewing clinician based on the infarct topography, size and pattern. The ‘embolic’ definitions referred to cases with multiple infarcts of similar age based on DWI. Lesion volumes were calculated following 3D segmentation using ITK-SNAP software. (Yushkevich et al., 2006). The presence of chronic white matter lesions was recorded and graded using the Fazekas score. (Fazekas et al., 1987).

Cases were classified into three groups, those with acute focal perfusion abnormality consistent with lacunar stroke on blinded assessment of parametric perfusion maps (Obvious perfusion lesion), those with perfusion abnormality only evident when viewed alongside follow-up DWI (Subtle perfusion lesion), and those without perfusion deficit despite follow-up DWI lesion (No perfusion lesion).

### 2.7 Statistical analysis

Statistical analysis was performed using Python 3.11 libraries; pandas, scikit-learn, statsmodels, numpy and matplotlib. Comparison between groups was performed using Chi-Squared tests for proportions. Shapiro-Wilks tests were used to determine normality and t-tests for normally distributed continuous data, while Mann-Whitney tests were used for non-normally distributed continuous variables. Where non-parametric tests were used, medians were reported rather than means. Ordinal logistic regression models were fit to determine impact of lesion visibility on functional outcomes.

### 2.8 Data availability

Individual patient data from INSPIRE is not publicly available. Individual data can be shared with partners following individual transfer agreements upon request with the corresponding author.

## 3 Results

**Fig. 1:**
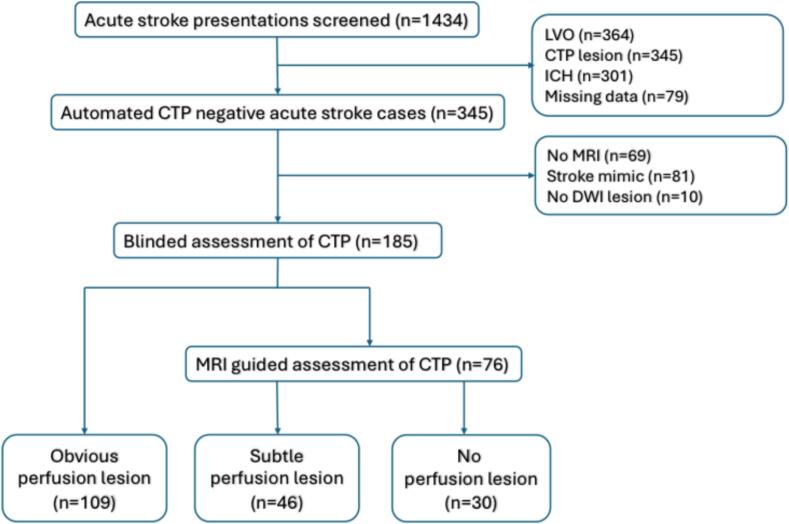
Study inclusion diagram – LVO = Large vessel occlusion, ICH = intracerebral haemorrhage, CTP = CT perfusion, DWI = diffusion weighted imaging.

Retrospective review of 1434 acute stroke emergency department presentations to two comprehensive stroke centres in metropolitan and regional New South Wales, identified a subset of 345 acute stroke presentations with no culprit vessel occlusion or algorithm detected core-penumbra perfusion lesion. Sixty-nine cases were excluded due to lack of follow-up DWI imaging, 81 due to non-stroke diagnosis, and 10 cases had no lesion on follow-up DWI (Fig. 1). The median CTP slice thickness was 1.25 mm (0.5 – 3 mm).

A total of 183 cases with confirmed acute stroke on follow-up MRI were included in the final analysis. 107 (58 %) cases had a perfusion lesion compatible with the clinical syndrome identified on CTP by clinicians blinded to DWI results (Obvious perfusion lesion). A further 36 cases (total 143, 78 %) had an area of CTP abnormality identified when viewed alongside coregistered follow-up MRI (Subtle perfusion lesion). There were 40 cases (22 %) where no focal abnormality was identified on CT that corresponded with the final infarct location on MRI (No perfusion lesion) (Table 1).

**Table 1:** Frequency of CT perfusion lesions.

CTP Lesion	N	Frequency
Obvious	107/183	58 %
Any (Obvious + Subtle)	143/183	78 %

**Fig. 2:**
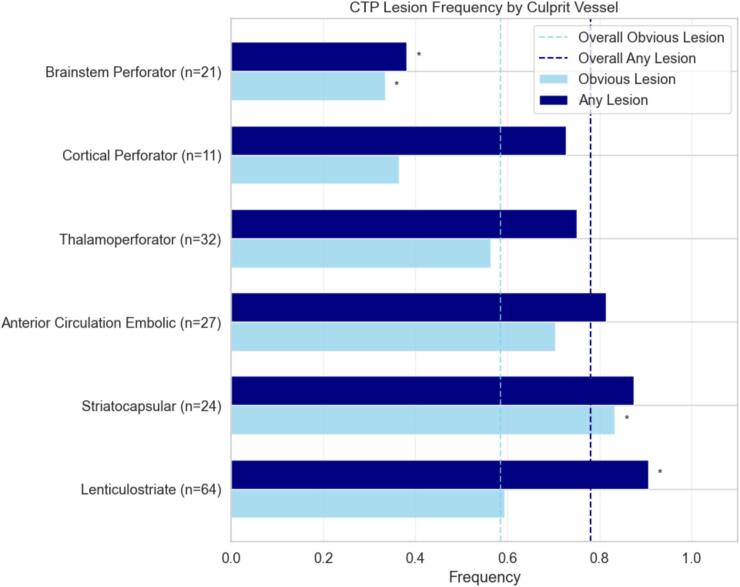
CTP lesion frequncy by Culprit Vessel – Frequency of ‘Obvious’ or ‘Any’ (obvious + subtle) perfusion lesions by culprit vessel compared to overall frequency indicated by dashed lines (obvious lesions and any lesion). (*) indicates significant difference compared with overall frequency (p < 0.05).

Frequency of CTP lesions varied significantly by culprit vessel. Brainstem perforator territory lesions were the least likely to be detected, with only 33 % identified as obvious and 38 % as subtle CTP lesions, both significantly lower than the overall frequency (p = 0.036 and p = 0.0002, respectively). In contrast, striatocapsular infarcts were more likely to present as obvious perfusion lesions compared to the overall cohort (83 % vs. 58 %, p = 0.02). Lesions involving lenticulostriate perforators were most frequently identified retrospectively as subtle perfusion abnormalities (90 % vs. 78 %, p = 0.026) (Fig. 2).

Cases with obvious versus subtle perfusion lesions showed similar characteristics overall, though obvious cases had larger final DWI volumes (2 ml vs 1.1 ml, p = 0.02) and were less severe clinically (Median NIHSS 3 vs. 5, p = 0.002) (Table 2, Fig. 4).

**Table 2:** Characteristic of obvious vs subtle perfusion lesion cases.

Variable	Obvious Lesion	Subtle Lesion	p-value	
Age − median (IQR)	67.0 (56.0–76.0)	68.5 (58.2–79.0)	0.66	
Baseline mRS − median (IQR)	0 (0–0)	0 (0–0)	0.64	
NIHSS − median (IQR)	5 (3–8)	3 (2–5)	0.002*	
Onset to CTP (h) − median (IQR)	4.5 (2.1–10.0)	5.0 (2.2–11.8)	0.49	
DWI Volume (ml) − median (IQR)	2.0 (0.8–5.3)	1.1 (0.6–2.2)	0.02*	
Fazekas score – median (IQR)	1 (0–1)	1 (0–2)	0.098	
Sex − Male, n (%)	76 (69.7)	28 (60.9)	0.42	
Hypertension, n (%)	65 (59.6)	29 (63.0)	0.96	
Hypercholesterolemia, n (%)	40 (36.7)	21 (45.7)	0.48	
Diabetes, n (%)	23 (21.1)	13 (28.3)	0.54	
Previous Stroke/TIA, n (%)	15 (13.8)	7 (15.2)	0.99	
Ischemic Heart Disease, n (%)	17 (15.6)	7 (15.2)	1	
Smoker, n (%)	46 (43.0)	13 (28.3)	0.19	
Lacunar Syndrome, n (%)	91 (84.3)	39 (84.8)	1	
• Motor Symptoms, n (%)	46 (42.6)	22 (47.8)	0.77	
• Sensory Symptoms, n (%)	5 (4.6)	3 (6.5)	0.89	
• Mixed Symptoms, n (%)	32 (29.6)	9 (19.6)	0.40	
• Ataxic Hemiparesis, n (%)	3 (2.8)	2 (4.3)	1	
• Clumsy Hand Syndrome, n (%)	1 (0.9)	1 (2.2)	1	
Lysis, n (%)	7 (6.5)	6 (13.3)	0.47

**Fig. 3:**
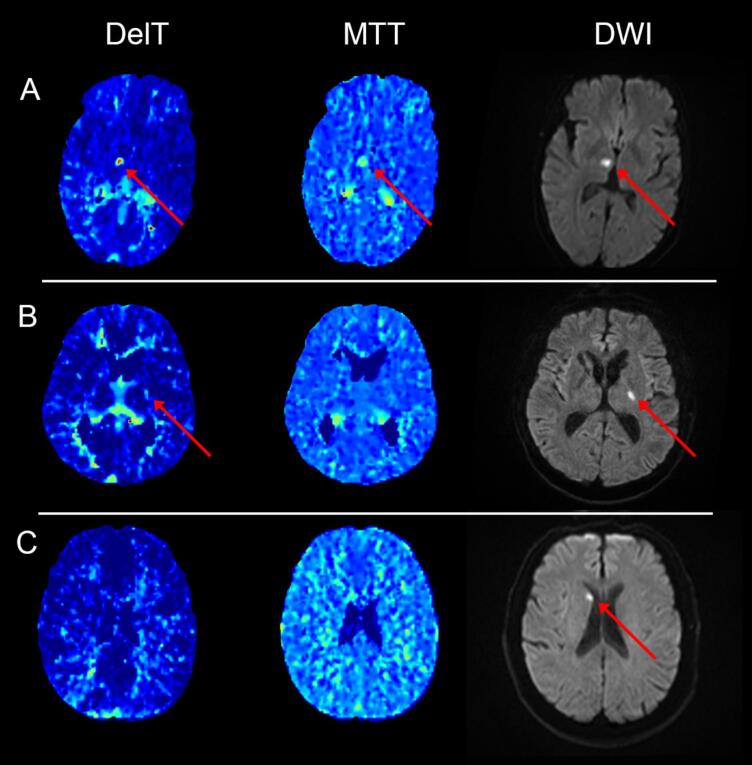
CTP Maps co-registered to DWI (A) – Obvious CTP lesion, (B) – Subtle CTP lesion, (c) – No CTP lesion. Lesion is highlighted in red across modalities. (For interpretation of the references to colour in this figure legend, the reader is referred to the web version of this article.)

**Fig. 4:**
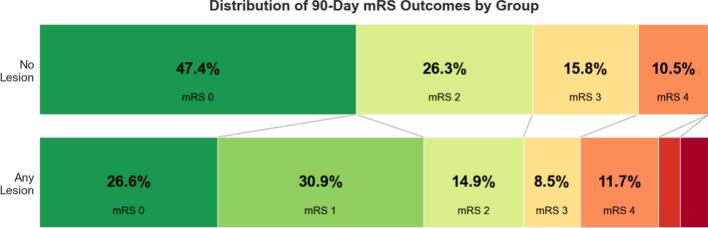
Distribution of 90-day mRS for cases with and without any perfusion lesion on acute CTP. Rates of mRS 5 and 6 in the “Any Lesion” group 3.2 and 4.3% respectively.

Compared to cases with normal perfusion on baseline CTP, those with any perfusion lesion (obvious or subtle) were less likely to have baseline disability (mRS > 0 27.5 % vs. 40 %, p = 0.013), and more likely to have a higher NIHSS (4 vs 2, p = 0.005) and larger final infarct volume on DWI (1.8 ml vs 0.7 ml, p < 0.001). Cases with perfusion lesions had baseline scanning significantly earlier than those with normal CTP (4.5 h vs 8.4 h, p = 0.033) (Table 3).

**Table 3:** Characteristics of CTP positive and negative cases.

	Perfusion Lesion	No Perfusion Lesion	p-value
Age − median (IQR)	67 (57–76)	74 (62–77)	0.077
Baseline mRS − median (IQR)	0 (0–0)	0 (0–2)	0.005*
• mRS > 0	25 (17.5)	12 (40)	0.013*
NIHSS − median (IQR)	4 (2–7)	2 (1–6)	0.005*
Onset to CTP (h) − median (IQR)	4.5 (2.1–10.8)	8.4 (3.0–17.1)	0.033*
DWI Volume (ml) − median (IQR)	1.8 (0.8–4.1)	0.7 (0.3–1.4)	<0.001*
CT z-axis coverage (mm) – median (IQR)	120 (111–160)	111 (100–140)	0.005*
Fazekas score – median (IQR)	1 (0–1)	1 (0–2)	0.219
Sex − Male, n (%)	99 (66.4)	16 (53.3)	0.247
Hypertension, n (%)	92 (61.7)	21 (70.0)	0.517
Hypercholesterolemia, n (%)	58 (38.9)	13 (43.3)	0.806
Diabetes, n (%)	34 (22.8)	12 (40.0)	0.083
Previous Stroke/TIA, n (%)	23 (15.4)	8 (27.6)	0.190
Ischemic Heart Disease, n (%)	24 (16.1)	6 (20.7)	0.740
Smoker, n (%)	53 (36.3)	12 (41.4)	0.759
Lacunar Syndrome, n (%)	125 (83.9)	24 (82.8)	1
• Motor Symptoms, n (%)	67 (45)	8 (27.6)	0.126
• Sensory Symptoms, n (%)	8 (5.4)	3 (10.3)	0.551
• Mixed Symptoms, n (%)	38 (25.5)	6 (20.7)	0.753
• Ataxic Hemiparesis, n (%)	6 (4)	0 (0.0)	0.591
• Clumsy Hand Syndrome, n (%)	2 (1.3)	0 (0.0)	1
Lysis, n (%)	14 (9.5)	1 (3.3)	0.453

Clinical outcomes did not differ significantly between groups, with high rates of good and excellent outcomes in both groups. There was a trend towards a higher rate of ‘perfect’ outcome in those without any perfusion lesion (mRS 0, OR 3.92, 95 %CI 0.97–15.84), though this did not reach statistical significance, and overall ordinal mRS shift analysis also did not demonstrate significant difference (Fig. 3).

## 4 Discussion

In this large, multicentre study of CT perfusion (CTP) imaging in patients with acute lacunar stroke, we demonstrated that perfusion abnormalities were present in 78 % of cases when CTP was reviewed in conjunction with follow-up DWI. However, only 58 % of cases had lesions identifiable on initial review (blind to DWI), highlighting the limited sensitivity of CTP in its current form when used as an acute diagnostic tool. Lesions not detected on CTP were significantly smaller, more frequently due to brainstem perforator occlusion, and associated with milder clinical symptoms, suggesting that the location and volume of affected tissue are major determinants of visibility on CTP maps.

Our findings align with and extend previous studies reporting variable sensitivity of CTP in lacunar stroke, which range from 0 to 62.5 % (Garcia-Esperon et al., 2021, Das et al., 2015, Rudilosso et al., 2015, Benson et al., 2016, Cao et al., 2016). By incorporating co-registered DWI to guide retrospective interpretation, we were able to separate limitations of the imaging modality from human interpretative error. This approach revealed that a considerable proportion of lacunar infarcts do produce perfusion abnormalities, albeit subtle ones that may be difficult to detect amidst the inherent noise in the subcortical regions on perfusion maps.

Cases without any identifiable perfusion abnormality on acute CTP were generally less severe, with lower NIHSS and smaller DWI lesion volumes. These characteristics may reflect cases with very distal perforator occlusion or early spontaneous reperfusion. Patients with longer onset-to-scan intervals were more likely to have normal perfusion on CTP, raising the possibility that some lesions may have spontaneously reperfused by the time of imaging. This hypothesis is consistent with pathological descriptions of preserved feeding arteriole patency in some lacunar infarcts (Fisher, 1979). These findings may have implications for treatment decision-making, particularly regarding the role of reperfusion therapy in cases without demonstrable perfusion deficits. Recent studies have not supported the use of IV thrombolysis in non-disabling stroke with a proven vessel occlusion or perfusion deficit, however these studies did not include lacunar stroke (Coutts et al., 2024). We demonstrated a lower proportion of milder stroke in cases with perfusion lesions. These ‘mild’ cases are often not treated with thrombolysis as the risks may outweigh potential benefits. Disabling strokes poorly captured by NIHSS, such as significant visual disturbance or aphasia, are far less likely to occur in lacunar stroke. The utility of IV thrombolysis in lacunar stroke is an area of ongoing research, one that may benefit from improved acute phenotyping of cases.

Cases with higher pre-stroke disability scores (mRS) were more likely to have ‘normal’ CTP imaging. These cases may reflect a greater burden of cerebral pathology (such as white matter disease, cortical atrophy or previous infarcts), which may increase the finding of chronic diffuse white matter hypoperfusion, making detection of the new acute lesion extremely difficult (Bivard et al., 2016). While our study was underpowered to detect sub-group differences, there was a trend to a higher proportion of previous stroke, and higher burden of white matter disease imaging markers in the patients with pre-stroke disability.

Technical limitations may further impair CTP’s utility in small vessel stroke. The signal-to-noise ratio of CTP is limited compared to MRI, and this limitation is amplified in deep or infratentorial regions by beam-hardening artefact and anatomical complexity. Subcortical lacunar perfusion lesions are often small and poorly contrasted against surrounding tissue due to the lower signal to noise in the white matter, making detection difficult even for experienced readers. Brainstem perforator strokes were particularly under-detected in our cohort, highlighting a significant limitation of CTP, and CT in general in this anatomical region. Improvements in scanner technology, such as increased field of view, detector sensitivity and improved post-processing techniques, show promise for improving image quality in the posterior fossa (Guziński et al., 2016).

The performance of CTP also depends on acquisition parameters, processing software, and clinician expertise. All scans in our study were processed using MIStar software at the highest available resolution without downsampling, and images were reviewed by experienced stroke neurologists. While this approach maximizes diagnostic yield, it may not reflect real-world practice in primary stroke centres, where imaging is often interpreted by generalists or non-specialist radiologists. Moreover, differences between commercially available software packages (e.g., MIStar vs. RAPID) may contribute to variability in lesion detection, as previously reported (Bivard et al., 2013).

Despite these limitations, our results highlight the evolving potential of CTP in small vessel stroke. Technological advances, including modern CT scanners, improved perfusion algorithms, and automated processing, are steadily improving image quality. Acknowledgment of the potential of CTP in diagnosing lacunar stroke may inform radiology and stroke training development to incorporate modules on interpretation of CTP parametric maps to identify lacunar lesions. Emerging machine learning approaches offer promising avenues for enhancing sensitivity and specificity by distinguishing true perfusion abnormalities from background noise (Khalifa and Albadawy, 2024).

Limitations of our study include the retrospective design and selection bias toward patients who underwent follow-up MRI, which may exclude those with very mild or very severe strokes. The sample was drawn from comprehensive stroke centres in Australia, where CTP is routinely performed and interpreted by stroke specialists, limiting generalizability to lower-resource settings. Additionally, our study was not powered to assess differential outcomes by infarct location or treatment subtype, and future prospective studies should explore these associations further. Our methodology was not designed to measure the clinical accuracy of CTP for lacunar stroke as we excluded DWI negative cases. While our reported frequencies of 58 and 78 % are analogous to a measure of sensitivity, we are unable to comment on specificity as we intentionally excluded false positives from our analysis.

In summary, CTP perfusion abnormalities are present in the majority of lacunar strokes, especially when interpreted with reference to follow-up imaging. However, presence of a discernible lesion is limited by lesion size, scan timing, anatomical location, and imaging noise. CTP-negative lacunar infarcts appear to represent a milder clinical subgroup, who may not require thrombolytic therapy. Further research should explore methods for improving lesion detection and investigate the prognostic and therapeutic implications of perfusion status in small vessel stroke.

## CRediT authorship contribution statement

**James O. Thomas:** Writing – review & editing, Writing – original draft, Visualization, Validation, Methodology, Investigation, Formal analysis, Data curation, Conceptualization. **Milanka Visser:** Validation, Software, Data curation. **Carlos Garcia-Esperon:** Writing – review & editing, Supervision, Methodology, Conceptualization. **Neil J. Spratt:** Writing – review & editing, Supervision, Conceptualization. **Dennis Cordato:** Writing – review & editing, Supervision. **Cecilia Cappelen-Smith:** Writing – review & editing, Supervision. **Longting Lin:** Writing – review & editing, Supervision, Methodology, Formal analysis, Conceptualization. **Mark W. Parsons:** Writing – review & editing, Supervision, Methodology, Conceptualization.

## Funding

Nil declared.

## Declaration of competing interest

The authors declare that they have no known competing financial interests or personal relationships that could have appeared to influence the work reported in this paper.

## Data Availability

Individual patient data from INSPIRE is not publicly available. Individual data can be shared with partners following individual transfer agreements upon request with the corresponding author.
